# Report of Two Cases of Lithotomy

**Published:** 1875-11

**Authors:** E. A. Jelks

**Affiliations:** Quitman, Brooks County, Ga.


### REPORT OF TWO CASES OF LITHOTOMY.

By E. A. JELKS, M.D., of Quitman, Brooks County, Ga.

Mr. A. J. Williams, of Taylor county, Florida, about forty-five years of age, of sanguine temperament, weight in health two hundred pounds. For years he had been troubled with difficult micturition, and by the advice of a neighbor, supposing he had stricture of the urethra, he had been in the habit of using a sweet-gum stick to dilate the tract, and during the operation some weeks previous he said it slipped into the bladder. On examination with the sound a foreign body of considerable size was detected in the bladder. In consequence of continual suffering from some vesical trouble, his condition was such that I advised an operation as soon as he could have it performed. To this he consented, and on the 14th of January, 1869, with the assistance of Drs. Briggs, Wilkinson, and Polhill, the former holding the staff, I performed the lateral operation, extracting two calculi about the size and shape of a pullet's egg cut through the long diameter. On recovery from the chloroform, Williams inquired for the sweetgum stick, and when informed that none was to be found, he was not a little astonished—having been under the delusion that the stick had slipped into the bladder. He required but little after treatment, and was up about the room in one week from date of operation. Since then his health has been excellent.

The second case, little Jimmy Price, a colored boy of our place, eight years of age, from whom I removed a urethral calculus two years ago. Since then he has suffered more or less with vesical trouble, and lately his sufferings have been almost unbearable. On the 10th day of August last, with the assistance of Drs. Briggs, Wilkinson, and Carswell, and Dental Surgeon Snow, the first holding the staff, the last administering chloroform, I operated, performing, as in the first case, the lateral operation. Had but little difficulty in getting into the bladder, but the stone was of such large dimensions that it was difficult to remove. Using what force I thought prudent and failing, I handed the forceps to Dr. Briggs, whose good suggestions have so often led me safely through the breakers. He tried for some minutes to extract the stone, when he gave it up and suggested a drill, as we had no crusher. I however took the forceps, and on introducing them again decided it would be best to try the drill, when Dr. Carswell requested me to let him have the forceps. I gave them to him, and by almost super-human exertions he succeeded in pulling it through. The little fellow suffered greatly from the shock and possibly from the effects of chloroform, and it was sometime before reaction was established. He, however, rallied, improved steadily, and is now running about the yard, the wound being entirely healed.

When we consider the size of the prostate in a lad of eight summers, and the size of the stone in this case, is it not a little remarkable that there was no urinary infiltration? Both of these operations were performed with an ordinary English bistoury that did me good service during the late war between the States, and a staff with a groove on the convex surface, not being able to get one with a groove between the side and convex surface. I think, however, this is of little moment, as the knife can be lateralized sufficiently with the kind used. This latter stone was very rough and hard, measuring six inches one circumference and five the other, weight $2\frac{3}{4}$ ounces, composition phosphate of ammonia and magnesia. The first we did not analyze.

